# Secondary Bleedings in Oral Surgery Emergency Service: A Cross-Sectional Study

**DOI:** 10.1155/2018/6595406

**Published:** 2018-06-03

**Authors:** Sebastian Igelbrink, Stefan Burghardt, Barbara Michel, Norbert R. Kübler, Henrik Holtmann

**Affiliations:** ^1^Clinic for Oral and Maxillofacial Surgery, University of Münster, Albert-Schweitzer-Campus 1, 48149 Münster, Germany; ^2^Clinic for Oral and Maxillofacial Surgery, University Clinic of Duesseldorf, Moorenstr 5, 40225 Duesseldorf, Germany; ^3^Doctor's Practice for Oral and Maxillofacial Surgery, Uhlstraße 97, 50321 Brühl, Germany

## Abstract

**Introduction:**

Bleeding after dental surgery is still a common cause for emergency presentation in patients using anticoagulants. Our aim was to analyze pertinent characteristic features on the one hand and to bare existing problems in handling on the other.

**Materials and Methods:**

The study included 76 patients. We documented basic data, anticoagulant medication, type of surgery, and tooth socket sutures in respective patients.

**Results:**

The vast majority of patients took a coumarin derivative (41) and acetylsalicylic acid (27). Nine (12%) of the patients had to be hospitalized due to ongoing bleeding despite local haemostyptic steps and/or circulatory dysregulation. Most patients could be successfully treated in outpatient settings. No statistically significant correlation between bleeding, level of INR value, number of extracted teeth, and sewed alveoli could be shown. Sixty-five percent of cases with tooth extractions did not have suture of tooth sockets. Eighty-seven percent of the patients denied being informed about possible self-treatment options by their surgeon/dentist, and none of the patients got presurgical-fabricated bandage plate(s).

**Conclusions:**

Patients taking coumarin derivative currently, furthermore, represent the biggest anticoagulant after-bleeding group in dentoalveolar surgery. The major part of after-bleedings (90%) can be handled in an outpatient setting with simplest surgical interventions. Unfortunately, the biggest part of the patient collective got no suture, no prefabricated dental bandage plate(s), and no explanation by their dentist how to handle in case of after-bleeding. Therefore, dental practitioners should furthermore get enlightenment on how to prevent after-bleeding situations.

## 1. Introduction

Secondary bleedings after dental surgeries can lead to emergency presentation. Influencing factors include medication, the performed surgery, and patient factors. With regard to the management of perioperative coagulation, for example, the guidelines of the European Society of Cardiology (ESC) and the European Society of Anaesthesiology (ESA) state that noncardiac operations can be carried out safely at an INR of <1.5 [1]. According to the German Cardiac Society, for dentoalveolar surgery, an INR of 1.8–2.0 may be acceptable should a patient's thromboembolic risk require it [2].

Numerous papers on the issue of secondary bleeding after oral surgery [3–13] can be found in the literature. Recommendations for perioperative bleeding management especially advocate for suture of the tooth sockets following exodontia [14, 15].

However, all these works have some limitations. Some do not break down, for which surgeries were performed [11, 16]. Others focus largely or exclusively on tooth extractions [3, 5, 7–10, 13, 17]. Also, some papers do not mention their surgical management of secondary bleedings [12]. Some works mainly included patients operated on in university settings, thus reducing their validity for the nonacademic sector considerably [5, 13]. One study concludes that there is heterogeneity and poor comparability within the present literature [6].

Therefore, our study aimed to answer the following questions in particular:

- Which is the mean INR value in patients using phenprocoumon medication/vitamin K antagonists? (In Germany, physicians typically prescribe phenprocoumon rather than warfarin as a coumarin derivative.) In after-bleeding, is there a statistically significant difference of the INR value between cases with single- versus multiple-teeth extractions?
- Do tooth-extracted patients whose alveoli were sewed over have higher INR values than patients whose alveoli were not? Are all anticoagulant patients regularly sewed over? Do they possess prefabricated bandage plates?
- Does an ongoing medication with acetylsalicylic acid play numerous roles concerning anticoagulant patients and bleeding after dentoalveolar surgery?
- What is the actual role in dentoalveolar secondary hemorrhage of the new direct oral anticoagulants (DOACs) in comparison to the classical ones?
- How extensive is the needed therapy in after-bleeding cases, and what is the percentage of patients with an inpatient treatment? What is the reason for inpatient treatment in dentoalveolar after-bleeding patients?
- Are patients well informed about easy and appropriate measures which they can do on their own against bleeding by their medical practitioner who did dentoalveolar surgical intervention?

## 2. Materials and Methods

### 2.1. Study Aim, Design, and Setting

The aim was to analyze characteristic features in patients with emergency presentation due to bleedings following dental surgery. We designed a cross-sectional study at the University Clinic of Duesseldorf. The study was conducted after registration with the Ethics Committee of the Medical Faculty of the Heinrich Heine University of Duesseldorf.

### 2.2. Patients

All patients who presented to the Department of Oral and Maxillofacial Surgery, University Clinic of Duesseldorf, with secondary bleedings following dental surgery and use of anticoagulants were eligible. We comprised patients into the study over a period of 12 month (April 2015–March 2016).

We excluded patients with concomitant haemostaseologic or hematological diseases and those presenting with (intraoral) bleedings from tumor arrosion.

### 2.3. Data and Statistical Analysis

Following informed consent, we recorded demographics, surgical history (type of oral surgery performed, setting, and technique of anesthesia), anticoagulant medication, and its last intake. In cases with phenprocoumon medication, we also recorded the initial INR value in patients' presentation. We noted the number of extracted teeth and whether the pretreating surgeon had sewed over the alveoli. In addition, we asked the patients by questionnaire and recorded whether they were informed about actions they can do on their own in case of bleeding, the reason for dentoalveolar surgery, and whether the patients were in possession of/wore presurgical dentist-fabricated bandage plate(s). Finally, we documented the ensuing surgical procedures performed in our clinic, the duration and reason of/for any inpatient stay (if necessary), and whether erythrocyte concentrates had to be given.

Statistical analysis was done using Excel 2013 (Microsoft Corporation, Redmond, Washington, USA). We calculated *t*-tests and selected the appropriate subtype after performing an F-test to evaluate the variance of the compared groups. Confidence intervals were calculated at 95% level. Student's *t*-test was chosen in case of a group size < 30. In case of a group size > 30, we assumed a normal distribution and calculated the confidence intervals accordingly. We also calculated odds ratios to compare groups. Finally, Cohen's *d* or Cramer's V for groups of different sizes are given to describe strengths of association between characteristics [18].

## 3. Results

### 3.1. Participants

Seventy-six patients, 51 men (67%) and 25 (33%) women, were included. The mean age of these patients was 67.5 years.

### 3.2. Anticoagulation

**Figure 1:**
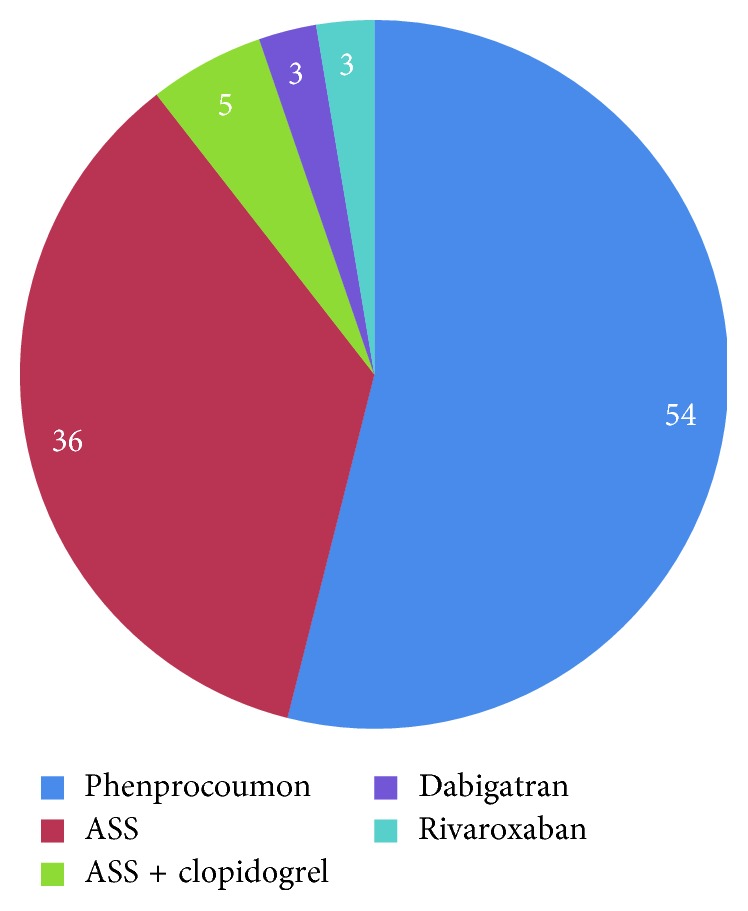
Percentages of primary anticoagulant substances taken.

Forty-one patients stated to be taking phenprocoumon, 27 acetylsalicylic acid, 4 acetylsalicylic acid combined with clopidogrel, 2 rivaroxaban, and 2 dabigatran (Figure 1). For the indications for intake of anticoagulation, see Table 1. The mean duration of drug holiday in patients with phenprocoumon medication was 2.6 days before surgery; however, 19 patients had continued to take phenprocoumon.

### 3.3. Surgical History

Sixty-eight of the patients had undergone tooth extraction. Eighteen patients got their extraction(s) isolated due to caries, 43 due to a combination of caries and chronic parodontitis, and 7 due to isolated acute/chronic parodontitis (latter data: patient's self-assessment). In 2 of the remaining patients, scaling and root planing was performed, and apicoectomy was performed in 6 patients.

In the phenprocoumon patients, 8 patients had a single tooth extracted, 31 patients had multiple teeth, and 2 patients had undergone apicoectomy (Table 2). 66 (87%) of the operations had been performed in a practice, 10 (13%) of the operations in a clinic. An average of 2.9 teeth had been extracted in the exodontia patients. In only 24 (35%) of the patients after tooth extraction, the pretreating surgeons/dentists had sewed over the alveoli. In the subgroups of patients with phenprocoumon, the extraction sockets had been sewed over in 15 cases (38%) and in those with ASA medication in 6 cases (27%) (Table 3).

Concerning our question if patients were well informed about easy and appropriate measures which they can do on their own against bleeding by their medical practitioner who did dentoalveolar surgical intervention, 87% of the patients negated this. Furthermore, none of the presented patients declared the possession of or wore (a) presurgical-fabricated bandage plate(s).

### 3.4. INR

In the 41 patients of the phenprocoumon group, the mean INR was 1.9 (1.78, 2.02). The lowest INR was 1.3 and the highest INR was 3.0.

In the phenprocoumon bleeding group, the INR did not differ statistically significantly with regard to the presence of sutures of the alveoli as well as extraction of one versus multiple teeth (*p*=0.31) (Table 4).

### 3.5. Further Treatment

Local ambulant treatment was sufficient in 67 (88 (81, 95) %) patients (bite swab with or without tranexamic acid, suture, and haemostyptic agent). Only 9 (12 (5, 19) %) of the patients had to be hospitalized due to isolated ongoing bleeding despite local haemostyptic steps (*n*=5) and/or circulatory dysregulation (*n*=3/*n*=1). 6 of these patients had taken phenprocoumon, 2 patients had taken acetylsalicylic acid, and 1 patient had taken clopidogrel + acetylsalicylic acid. The mean age of patients who had to be hospitalized was 68 (53.5, 82.8) years. The duration of the inpatient stay was 2.8 (2.3, 3.3) days on average. No case required the transfusion of erythrocyte concentrates.

### 3.6. Comparison of Patients with Consecutive Inpatient Admission with Outpatients

Patients after tooth extraction had to be admitted significantly less frequently (9% versus 38%, *χ*2 test = 0.02, and odds ratio = 0.16), but with very weak association (Cramer's V = 0.02). Patients who were hospitalized after tooth extraction showed no statistically significantly higher extraction number (4.2 versus 2.7. *p*=0.14). Similarly, phenprocoumon patients whose INR were not statistically significant were more likely to be hospitalized than other patients (*p*=0.49). Patients after periodontal surgery, on the other hand, had to be admitted statistically significantly more frequently (*p* < 0.001); however, as mentioned, there were totally only 2 periodontal cases (Table 5).

In all 9 patients who were admitted, we took impressions for wound covers. Those patients following exodontia and apicoectomy also received additional sutures. In the two periodontal cases, we applied periodontal dressing (Peripac®, Dentsply). For residual minor bleedings, tranexamic acid gauze was used as needed (4 cases).

## 4. Discussion

The mean INR of phenprocoumon patients was only 1.9 (1.78, 2.02). In particular, there was no statistically significant difference in INR values between patients with single-tooth extractions and those with multiple-tooth extractions. The statistical significance was closely missed (*p*=0.08); however, even if it had been met, the result would have had little effect as a *χ*2 test of 0.08 was shown. An explanation for this could be that lower values rarely lead to secondary bleeding, and higher values are rare due to bridging to heparine. There was also no statistically significant difference with respect to the INR (1.94 versus 1.88, *p*=0.31) concerning extraction patients whose alveoli were sewed and patients whose alveoli were not.

At the first sight, this result seems to suggest that neither single-tooth extractions nor sutures of the alveoli allow for higher tolerable perioperative INRs. However, this conclusion would be premature: the average INR in the basic population was not known, and the same was true for the proportions of patients with sutures of the alveoli and single-tooth extractions.

The percentage of patients who had to be admitted to the hospital was 12% [5, 19] (9 patients) with no indication to blood transfusion. This correlates to the current literature which shows that in most cases of bleeding, secondary to oral surgery easy measures are enough to stop the bleeding [16, 20]. On the other hand, the small proportion of patients needing inpatient stay shows that the recommendations for the ongoing use of anticoagulants during oral surgery seems to be appropriate [16, 21, 22].

The mean age of patients (68 years) probably represents the age group affected in industrialized countries with considerably ageing populations well. This is especially true when compared to the work of Pereira et al. from Brazil [9]. In his study, the mean age of patients with phenprocoumon was 49 years.

The gender ratio of 67 : 33% men to women is consistent with the data by Koertke (approx. 70% share of men) and Maegerlein (74%) regarding phenprocoumon medication after mechanical heart valve replacement [19, 23]. The patient sample is likely to be representative for the ambulatory sector as well because of the large catchment area of a university emergency department.

A Hawthorne effect on the part of pretreating surgeons and patients can be excluded because both groups were not aware of the study before a participant's presentation to our emergency room. Furthermore, the cross-sectional design makes this effect unlikely.

Considering the study collective at all, one could find further interesting facts: first thing was that patients using DOAC up to now do not play a numerous important roles in dentoalveolar after-bleeding up to now. We could only identify four patients using them in our collective. None of them had an inpatient stay. Contrary to this fact, coumarin-intake patients took the vast majority of all after-bleeding patients (41 patients). Additionally, as mentioned before, the highest percentage of patients reported that they were not informed properly about easy measures they could do on their own against after-bleeding (87%), and none of them possessed prophylactica bandage plates. Unfortunately, 2/3 of the after-bleeding patients had no suture. The fact that 1/3 had after-bleedings despite suture may have special reasons one could speculate for: ease of the suture material perhaps due to solid food, misleading preparation and mobilization of sore rand, increase of blood pressure due to postoperative pain, inadequate sewed wounds, and basic need for more protective measures than only suture (additional need for hemostytic agents and bandage plates) in cases of extended dentoalveolar surgery. Unfortunately, these data could not be obtained in this study. Further studies could pursue these conjectures.

For inpatient treatment, phenprocoumon also numerously played the most important role. For inpatient care, no blood transfusion or antagonization of anticoagulation was necessary, which fits to the literature published before [16].

Due to the study setting with patients who had had surgery alio loco in most cases and presented in an emergency, reliable bridging protocols could not be obtained. However, such data would have had to rely solely on possibly incomplete patient information in many cases.

The vast majority of our patients had undergone extractions due to a combination of caries and parodontitis or isolated parodontitis (50/68) with a presumable higher amount of granulation tissue before extraction. Additionally, patients after periodontal surgery had to be statistically more often treated inpatient. This could present a correlation between higher amounts of infection/granulation tissue and dentoalveolar bleeding risk. Nevertheless, these findings have to be interpreted with care due to the fact that the number of inpatient treatments was small, and the statements to the reason of initial surgical intervention was made by the patients themselves and not by the primary surgeon.

## 5. Conclusions

Bleeding secondarily to oral surgical interventions is still an important topic to talk about although numerous papers as well as national and international guidelines are written about that.

Our data reveal that about 90% of dentoalveolar after-bleedings could be handled in an outpatient setting with simplest surgical interventions (bite swab with or without tranexamic acid, suture, hemostyptic agent, and prefabricated bandage plate by a dentist/surgeon). Furthermore, all anticoagulant patients should always be given information about easy measures against after-bleeding before surgical intervention (antiseptic mouth rinse, soft costume, external cool packs, and bite swabs) and all patients should be sewed over within surgical intervention.

The possibility to handle mostly all patients in outpatient settings by simple measures underlines current literature findings which recommend no interruption of classical or direct oral anticoagulants. An interruption could lead to strong side effects like myocardial infarction or a stroke instead. Medication should be in its therapeutic range, and patients should closely be monitored. Furthermore, a precise time planning for dentoalveolar surgery is the most important thing for patients using DOAC [16, 21].

In addition, special attention should be put on vitamin K antagonist patients as they are the most numerous populations in secondary bleeding after dental surgery in our study collective and furthermore the biggest proportion of a small population needing inpatient treatment. One should handle therefore these patients with care and discuss any surgical step with their general practitioner.

Summarizing the data of the presented study, there is an ongoing need for more clarification for dental practitioners doing dental surgery for anticoagulant patients.

## Figures and Tables

**Table 1:** Reasons for anticoagulation (multiple entries were possible).

Stent: 10
Stroke: 5
Atrial fibrillation: 17
Valve replacement: 22
Myocardial infarction: 18
Thrombosis: 12
Others: 2

**Table 2:** Performed surgeries

Surgery	Number
Tooth extraction	68
Apicoectomy	6
Periodontal	2
Total	76

**Table 3:** Number (percentage) of extraction sockets with and without sutures in patients with respective anticoagulation.

	Suture	No suture
All	24 (35%)	44 (65%)
Phenprocoumon	15 (38%)	24 (62%)
ASA	6 (27%)	16 (73%)
ASA + clopidogrel	0 (0 %)	3 (100 %)
Dabigatran	1 (50%)	1 (50%)
Rivaroxaban	2 (100%)	0 (0%)

**Table 4:** INR values (phenprocoumon patients) with regard to sutures to the alveoli and extent of exodontia.

	Number	INR (mean)	Statistical test	Results
Sutures	15	1.94 (1.73, 2.15)^1^	One-sided *t*-test, equal variances/d_Cohen_	0.31/0.15
No sutures	24	1.88 (1.71, 2.04)		
Single-tooth extraction	8	2.08 (1.68, 2.47)	One-sided *t*-test, unequal variances/d_Cohen_	0.08/0.08
Multiple-teeth extraction	31	1.85 (1.72, 1.99)		

^1^95% confidence interval each.

**Table 5:** Statistical comparisons of patients who were admitted/were not admitted to the hospital.

	Admitted	Not admitted	Statistical test	Results
Total	9 (12 (5, 19)^1^%)	67 (88 (81, 95) %)	—	—
Mean age (years)	68	66	One-sided *t*-test, unequal variances/d_Cohen_	0.46/0.05
Surgical history				
Exodontia	6 (9%)	62 (91%)	Odds ratio/*χ*2 test/Cramer's V	0.16/0.02^2^
Number of extracted teeth (mean)	4.2	2.7	One-sided *t*-test, unequal variances/d_Cohen_	0.14/0.88
Periodontal surgery	2 (100%)	0 (0%)	*χ*2 test/Cramer's V	<0.001^2^/0.001
Apicoectomy	1 (17%)	5 (83%)	Odds ratio/*χ*2 test/Cramer's V	1.55/0.70^2^/0.10
Anticoagulation				
Phenprocoumon	6 (15%)	35 (85%)	Odds ratio/*χ*2 test/Cramer's V	1.83/0.41/0.07
INR (mean)	1.80	1.92	One-sided t-test, unequal variances/d_Cohen_/d_Cohen_	0.25/0.31
ASA	2 (7%)	25 (93%)	Odds ratio/*χ*2 test/Cramer's V	0.48/0.37/0.07
ASA + clopidogrel^3^	1 (33%)	3 (67%)		
Dabigatran	0 (0%)	2 (100%)		
Rivaroxaban	0 (0%)	2 (100%)		

^1^95% confidence interval each; ^2^expected values of the *χ*2 test too low → limited accuracy; ^3^due to very small number of cases, no further statistical testing was carried out.

## Data Availability

The data used to support the findings of this study are available from the corresponding author upon request.

## Abbreviations

DOAC: Direct (new) oral anticoagulant
ESC: European Society of Cardiology
INR: International normalized ratio.
